# Use of a cocktail of spin traps for fingerprinting large range of free radicals in biological systems

**DOI:** 10.1371/journal.pone.0172998

**Published:** 2017-03-02

**Authors:** Valérie Marchand, Nicolas Charlier, Julien Verrax, Pedro Buc-Calderon, Philippe Levêque, Bernard Gallez

**Affiliations:** 1 Louvain Drug Research Institute, Biomedical Magnetic Resonance Research Group, Université catholique de Louvain, Brussels, Belgium; 2 Louvain Drug Research Institute, Toxicology and Cancer Biology Research Group, Université catholique de Louvain, Brussels, Belgium; 3 Facultad de Ciencias de la Salud, Universidad Arturo Prat, Iquique, Chile; Albany Medical College, UNITED STATES

## Abstract

It is well established that the formation of radical species centered on various atoms is involved in the mechanism leading to the development of several diseases or to the appearance of deleterious effects of toxic molecules. The detection of free radical is possible using Electron Paramagnetic Resonance (EPR) spectroscopy and the spin trapping technique. The classical EPR spin-trapping technique can be considered as a “hypothesis-driven” approach because it requires an *a priori* assumption regarding the nature of the free radical in order to select the most appropriate spin-trap. We here describe a “data-driven” approach using EPR and a cocktail of spin-traps. The rationale for using this cocktail was that it would cover a wide range of biologically relevant free radicals and have a large range of hydrophilicity and lipophilicity in order to trap free radicals produced in different cellular compartments. As a proof-of-concept, we validated the ability of the system to measure a large variety of free radicals (O-, N-, C-, or S- centered) in well characterized conditions, and we illustrated the ability of the technique to unambiguously detect free radical production in cells exposed to chemicals known to be radical-mediated toxic agents.

## Introduction

**Fig 1:**
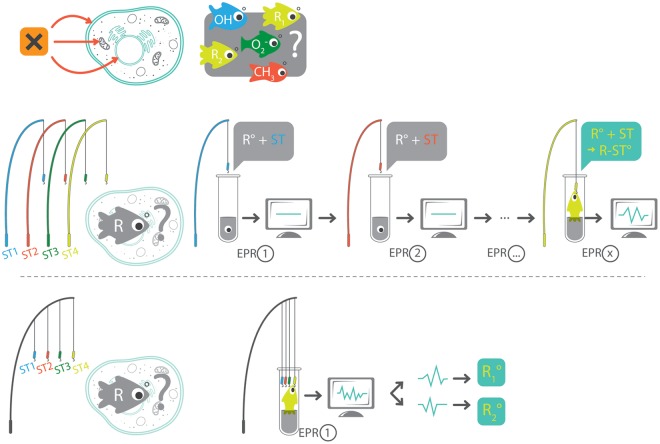
Pathway to demonstrate the involvement of free radicals in a toxicological process. Top: Hypothesis that a compound may lead to the production of free radicals in a biological medium. This process may involve various free radicals that can be produced extracellularly or in different cellular compartments. Middle: Traditional spin trapping pathway: the design of the experiment requires an *a priori* assumption regarding the nature of the free radical. Because the choice of spin-trap depends on its ability to react with the expected free radical or the expected localization of the site of production (intracellular or extracellular), this leads to a long iterative process. The hook needs to be adapted to each type of fish and its localization. Bottom: *Radicalomics*, using a cocktail of spin traps, reveals the presence of free radicals in one step, whatever the chemical nature of the free radical or the site of production. The EPR signature obtained can be deconvolved to characterize the likely radical involved. Comparison: Whatever the fish and its localization, it will be hooked after a single cast.

Reactive oxygen species (ROS), including free radicals, are considered to be harmful agents involved in the genesis of various pathologies, but are also important mediators in a range of biological and physiological processes [1]. Surprisingly, research into oxidative stress, oxidative damage, redox biology and antioxidants, is the subject of much controversy [1–3]. Leading researchers in the field generally consider that the inconsistencies published in the literature arise either from misidentification of the ROS involved in a process or from the uncertain causal link of the ROS with the biological consequence [1]. Support for the occurrence of oxidative stress in tissues is, therefore, sometimes based on the presence of end products of a sequence of events or on poorly validated biomarkers [2, 4], so that any conclusions drawn may be disputed. This limitation has stimulated recent research for new probes specific to a particular ROS or to ROS accumulating in a particular cell compartment [5, 6]. The term “ROS” encompasses a range of compounds that includes free radicals and other non-radical reactive species. To unequivocally identify the presence of a free radical, the method of choice is Electron Paramagnetic Resonance (EPR) spectroscopy using spin-trapping experiments. EPR is a magnetic resonance-based method that detects only species with unpaired electrons. To detect a short-lived free radical, a spin-trap, generally a nitrone, is added to a system to react with the free radical and thus forms a spin-adduct, a nitroxide radical that is longer-lived than the original radical [7, 8], and consequently more conveniently detected. The identity of free radicals can be inferred from the particular EPR spectrum using EPR constants, such as *g* values and hyperfine splitting constants. Although this method has been extended to simple sensitive immunoassays for detecting large radical molecules, such as DNA- or protein-radicals [9–11], EPR remains the gold standard for identifying the presence of radicals in small molecules. It is important to note that the classical EPR spin-trapping technique is a “hypothesis-driven” approach rather than a “data-driven” approach, because the design of the experiment requires an *a priori* assumption regarding the nature of the free radical. Hence, the choice of the spin-trap depends on its ability to react with the expected free radical or the expected localization of the site of production (intracellular or extracellular). Overall, experimental conditions are generally tuned to fit these *a priori* assumptions. This renders traditional spin-trapping experiments unpopular for rapid screening for possible free radical involvement in biological or toxicological processes because multiple experimental spin traps and conditions have to be performed sequentially (Fig 1). Therefore, it would be desirable to have a simpler and more general procedure that would facilitate a first exploratory step in the complex task of free radical detection, allowing the detection of a broader range of free radicals in biological systems. Rapid screening assays are indeed more and more needed to assist in the detection of radical-mediated oxidative stress without *a priori* knowledge of a specific mechanism.

**Fig 2:**
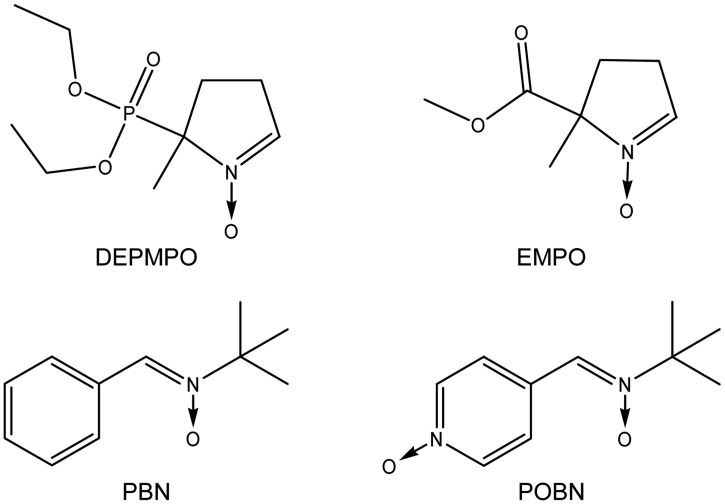
Chemical structures of the individual spin traps used in the proposed cocktail.

In the present work, we describe a simple assay based on a composition of selected spin-traps and a cell line sensitive to oxidative stress. Because there is no existing universal spin trap (able to trap and provide a signature for all types of free radical), we assembled a cocktail of different traps: N-tert-butyl-α-phenylnitrone (PBN), α(4-pyridil-1-oxide)-N-tert-butylnitrone (POBN), 5-diethoxyphosphoryl-5-methyl-1-pyrroline-N-oxide (DEPMPO), and 5-ethoxycarbonyl-5-methyl-pyrroline-N-oxide (EMPO) (Fig 2). The rationale for using this cocktail was that it would cover a wide range of biologically relevant free radicals and have a large range of hydrophilicity and lipophilicity (coefficient of partition between octanol and phosphate buffer from 0.15 to 10) in order to trap free radicals produced in different cellular compartments [8]. The selected spin traps offer a large variety in the EPR spectra of the spin adducts, without mutual interferences in order to discriminate as much as possible the free radicals involved. In the optimization procedure for the choice of the cocktail components, we discarded possible candidate spin traps, namely dimethyl-5,5 -pyrroline-N-oxide (DMPO), for which alternative reactions have been reported to lead to radical adduct artifacts in some cases [12, 13], and whose–OOH° adduct stability was lower than DEPMPO-OOH° [14, 15]; 2,2,4-trimethyl-2H-imidazole-1-oxide (TMIO) for which we found interfering background signals without free radical sources (data not shown) was also excluded. The assay is also composed of a cancer cell line, K562, commonly used to test the toxicity of molecules, and easy to grow in suspension. After preincubation of the spin traps and the K562 cells, the molecule to be tested is added and after further incubation at 37°C, the cellular suspension is measured by EPR spectroscopy.

As a proof-of-concept, we validated the ability of the system to measure a large variety of free radicals in well characterized conditions, and we illustrated the ability of the technique to unambiguously detect free radical production in cells exposed to chemicals known to be radical-mediated toxic agents. The corresponding spectra were simulated using published values of Hyperfine Coupling Constants (HFCC) and fitted to the experimental ones to further validate the method. This procedure, that we named *radicalomic* approach, involved the comparison of the EPR spectra obtained after adding the cocktail of spin traps to that obtained from a control or a treated medium (Fig 1).

The procedure should be considered as a rapid test, easily and conveniently allowing the detection of the production of free radical induced by a toxic molecule on a biological system, and to subsequently design more complex or more dedicated tests for further characterization of the radicals produced, if needed.

## Methods

### Reagents

Four spin traps were selected to cover a large range of lipophilicity and reactivity against the main families of radicals. DEPMPO and EMPO were obtained from Radical Vision (Marseille, France). POBN and PBN were purchased from Enzo-Alexis Biochemicals (Lausanne, Switzerland). Spin traps were dissolved in water, except for PBN which was dissolved in DMSO. Stock solutions (2 M) were stored at -80°C.

Chemicals for the radical generating systems (xanthine, xanthine oxidase, diethylenetriaminepentaacetic acid (DTPA), ferrous ammonium sulfate (Fe (NH_4_)_2_(SO_4_)_2_), hydrogen peroxide (H_2_O_2_), sodium sulfite, sodium dichromate, sodium azide and horseradish peroxidase) were purchased from Sigma Aldrich (St Louis, MO).

Toxic agents were chosen in order to produce radicals representative of the main types commonly observed (O, N, C or S-centered radicals). Menadione bisulfite, hydrogen peroxide, tert-butylhydroperoxide, phenylhydrazine, catalase (CAT) and superoxide dismutase-polyethylene glycol (PEG-SOD) were obtained from Sigma Aldrich (St Louis, MO).

### Spin trapping in free radical generating system

To generate superoxide anion radical, xanthine oxidase (1U/ml) was incubated with xanthine (1 mM) in PBS (pH 7.4, 100 mM) containing DTPA (500 μM) and 25 mM spin trap [16]. Hydroxyl radicals were generated from a Fenton system: Fe(NH_4_)_2_(SO_4_)_2_ (2mM) was added to a solution of 25 mM spin trap, 2 mM H_2_O_2_, and 1 mM DTPA in PBS (100 mM) [15, 17–19].

The sulfite radicals were generated and spin trapped by adding sodium sulfite (20 mM) and sodium dichromate (10 mM) into PBS containing 25 mM of the spin trap [15].

To generate methyl radicals, 10% v/v of DMSO was added to the Fenton system [15].

Azidyl radicals were generated by addition of sodium azide (30 mM) to a solution of 20 mM spin trap, DPTA (10 μM), H_2_O_2_ (350 μM) and horseradish peroxidase (1mg/ml) in PBS [20].

EPR spectra were recorded at room temperature using a Bruker EMX EPR spectrometer (Bruker, Germany) operating at 9.8 GHz, or a Bruker Elexys system, operated with the same parameters. The magnetic field center was 348.0 mT, with a Sweep Width of 15.0 mT (2048 pts). The microwave power was 3.19 mW; modulation frequency used was 100 kHz, modulation amplitude was 0.1 mT; the conversion time was 10.24 ms and the time constant was 10.24 ms. Fifteen scans were accumulated, and the experiments were made in triplicate. Spectra are the mean (Elexsys) or the sum (EMX) of the accumulated spectra. Consequently, intensity scales may differ between panels and figures.

### Cell culture conditions

The human leukemic cell line, K562, was purchased from the European Collection of Cell Cultures (ECACC, Salisbury, United Kingdom). These cells were selected because they are non-adherent and are commonly used to test the toxicity of chemicals. The cells were maintained in RPMI-1640 medium supplemented with 10% fetal calf serum, streptomycin 100 μg/ml, and penicillin 100 IU/ml (Gibco, Paisley, United Kingdom) at 37°C in a 95% air/5% CO_2_ atmosphere with 100% humidity.

### Spin trapping in cell cultures

Cells were incubated in RPMI-1640 medium at a concentration of 2.10^6^ cells/ml in a final volume of 0.5 ml. Cell suspensions were pre-incubated with individual spin traps (final concentration 50 mM), or the cocktail of spin trap (50 mM for each spin trap) for ten minutes at 37°C under 5% CO_2_. The toxic agent was then added directly to the medium and incubated for 10 minutes to generate radicals *in situ*. Based on data from the literature, hydrogen peroxide (5mM) was used to produce ^•^OH [21], tert-butylhydroperoxide (15mM) for alkoxyl radicals [22, 23], phenylhydrazine (5mM) for N centered radical [24]. Menadione (1mM final concentration) was used to produce the superoxide anion O_2_^•-^ and ^•^OH [25, 26].

K562 cells are chronic myeloid leukaemia derived cells [27] and are known to present both a higher basal production of ROS [28], and of detoxifying enzyme such as super oxide dismutase, or catalase [29]. Therefore they are much more resistant to toxic agents, hence the higher concentration of toxic needed to observe a toxic effect than usually required for other types of cell lines. The concentration of menadione (1 mM) was selected because no effect and no EPR signal were observed at 0.5 mM, whereas the concentration of 2 mM was the higher concentration giving an acceptable level of dead cells, below 10%.

In control experiments, when applicable, specific enzymes, such as PEG-SOD (100 U/ml) or CAT (100 U/ml), were used to degrade the toxin or the radical formed by the toxin and consequently suppress the EPR signal. Enzymes were added to the medium just before adding the spin trap.

For the EPR experiments in cellular systems, four hundred microliters of the cellular suspension were transferred into a quartz flat cell for aqueous samples (ER 160 FC-Q, Bruker, Rheinstätten, Germany). The cell was positioned in an X-band EPR Super High Q cylindrical resonator (ER4122SHQE, 10 mm diameter, Bruker, Germany) with its flat side perpendicular to the direction of the magnetic field. EPR spectra were measured at room temperature using a Bruker Elexys 540 spectrometer (Bruker, Germany) operating at 9.8 GHz. The magnetic field center was 348.0 mT, with a Sweep Width of 20.0 mT (512pts). The microwave power was 13 mW; modulation frequency used was 100 kHz, modulation amplitude was 0.1 mT; the conversion time was 20.48 ms and the time constant was 81.92 ms. Spectra were the mean of 30 scans.

### Simulation of EPR spectra

EPR spectra were simulated using Winsim [30] or Winsim 2002 from the P.E.S.T. (Public Electron Paramagnetic Resonance Software Tools) library of the National Institute of Environmental Health Sciences (NIEHS-NIH, NC, USA). Hyperfine coupling constants used for simulations were from the NIEHS-NIH database available on-line (Tables 1–5).

**Table 1:** Hyperfine coupling constants used for the simulation of EPR spectra for individual spin traps and for the combination of spin traps (cocktail). Reaction with hydroxyl radical.

OH°	N	H	P	area
POBN	15	1.73	-	-
PBN	16.04	3.25	-	-
DEPMPO	14	13.26	47.15	-
	14.49	21.48	46.65	-
EMPO	14.04	13.74	-	-
CKT	15.6	2.58	-	48.3
	14.86	21.28	-	16.6
	14.7	14.3	55.14	13

**Table 2:** Hyperfine coupling constants used for the simulation of EPR spectra for individual spin traps and for the combination of spin traps (cocktail). Reaction with azidyl radical.

N°	N	H	2e N	P	area
POBN	14.5	1.51	2.05	-	-
PBN	15.14	2.31	2	-	-
DEPMPO	13.85	12.48	2.82	45.78	89
	14.22	12.65	-	47.32	11
EMPO	14.05	13.01	-	-	50
	13.75	12.73	3.09	-	50
CKT	12.41	13.6	-	-	1.6
	14.56	13.61	3.3	-	16.6
	13.42	14.22	3.48	46.07	52.7
	13.99	13.49		47.87	29

**Table 3:** Hyperfine coupling constants used for the simulation of EPR spectra for individual spin traps and for the combination of spin traps (cocktail). Reaction with sulfite radical.

S°	N	H	P	area
POBN	14.5	1.5	-	-
PBN	14.85	1.89	-	-
DEPMPO	13.22	14.78	48.76	-
EMPO	13.52	15.01	-	-
CKT	13.18	14.71	48.8	52.95
	13.45	14.81	-	35.84
	14.21	21.45	46.21	11.21

**Table 4:** Hyperfine coupling constants used for the simulation of EPR spectra for individual spin traps and for the combination of spin traps (cocktail). Reaction with methyl radical.

CH3°	N	H	P	area
POBN	15.57	2.43	-	-
PBN	15.44	2.64	-	-
DEPMPO	15.11	22.11	47.51	-
EMPO	15.33	22.04	-	-
CKT	14.96	2.57	-	7.1
	15.79	2.65	-	25.7
	15.04	21.97	47.44	44.15
	15.18	21.75	-	23

**Table 5:** Hyperfine coupling constants used for the simulation of EPR spectra for individual spin traps and for the combination of spin traps (cocktail). Reaction with hydroperoxyl radical.

OOH°	N	H	P	area
POBN	15.93	2.7	-	-
PBN	15.89	3.27	-	-
DEPMPO	13.13	10.9	49.56	-
EMPO	13.2	11.98	-	52
	13.1	9.84	-	48
CKT	13.15	11.02	49.51	61.15
	13.29	11.8	-	21.46
	13.29	9.39	-	17.4

## Results and discussion

**Fig 3:**
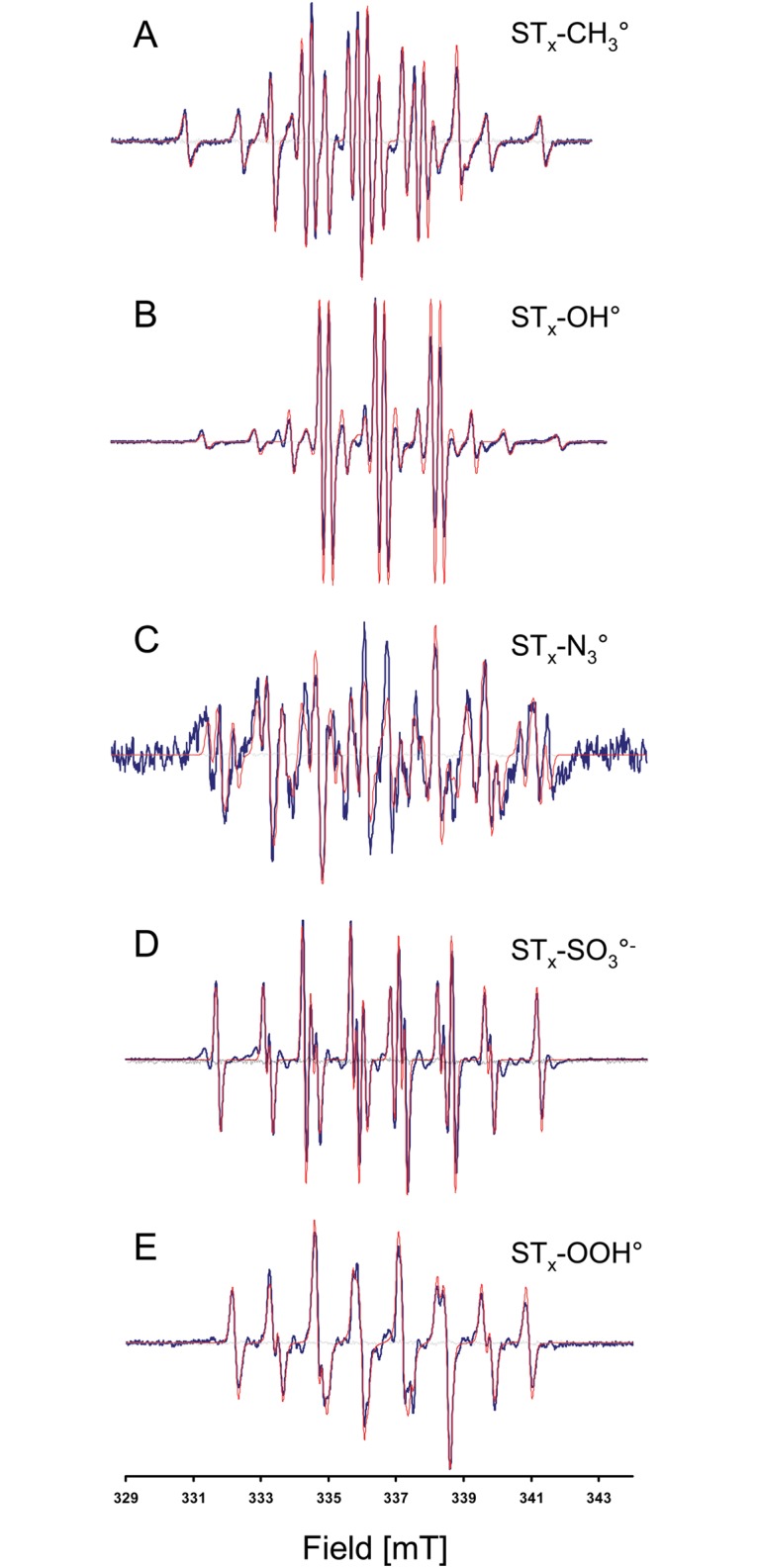
EPR spectra from the cocktail in the presence of different free radical generating systems. In Black, experimental spectra. In red, simulated spectra (using parameters described in Table 1). In grey, control experiments without generating system. A: methyl radical, B: hydroxyl radical, C: azidyl radical, D: sulfite radical, E: superoxide anion radical.

We first tested the ability of the cocktail of spin traps to give an EPR signature for biologically relevant free radicals produced by well-defined enzymatic or chemical reactions. Oxygen-centered radicals, hydroxyl radical and superoxide anion were produced by the Fenton reaction and a combination of xanthine and xanthine oxidase, respectively; a nitrogen-centered radical (azidyl radical) was produced by using azide, H_2_O_2_ and peroxidase; a carbon-centered radical (methyl radical) was produced using a Fenton system in dimethyl sulfoxide; a sulfur-centered radical (sulfite radical) was produced using sulfite in dichromate. The cocktail of spin traps reacted with all types of free radical, providing an EPR signature that was different from that of the controls (cocktail of spin traps without the generating system), and that was unique to and selective for the free radical produced (Fig 3). In these very simple assays, in which there was no restriction of access of the spin traps to the site of production of the radicals, all individual spin traps gave a spin adduct with an EPR signature compatible with the corresponding simulated composite spectra (S1–S5 Figs). The computed HFCC used to simulate the cocktail were in close agreement to constants published in the literature (Table 1) resulting in simulated spectra consistent with experimental spectra (Fig 3).

**Fig 4:**
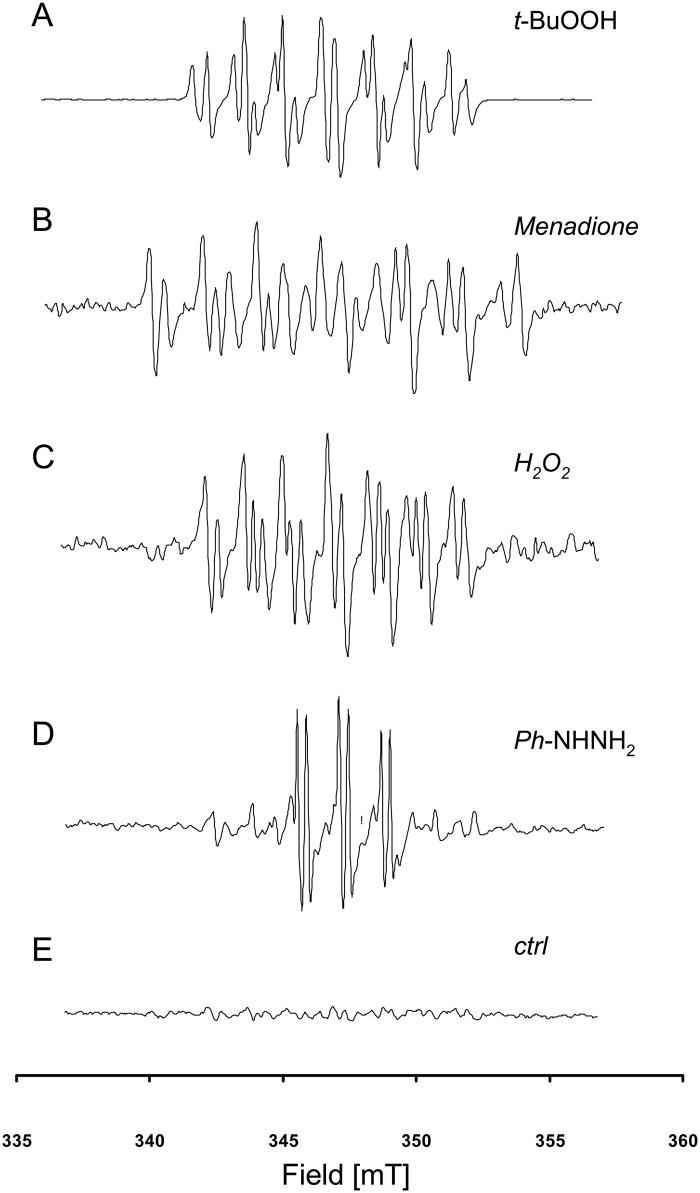
EPR spectra of K562 cells exposed to selected toxic agents. A: Tert-butylhydroperoxide, B: Menadione, C: Hydrogen peroxide, D: Phenylhydrazine. E: control experiment (K562 cells in the presence of cocktail without toxic agent).

**Fig 5:**
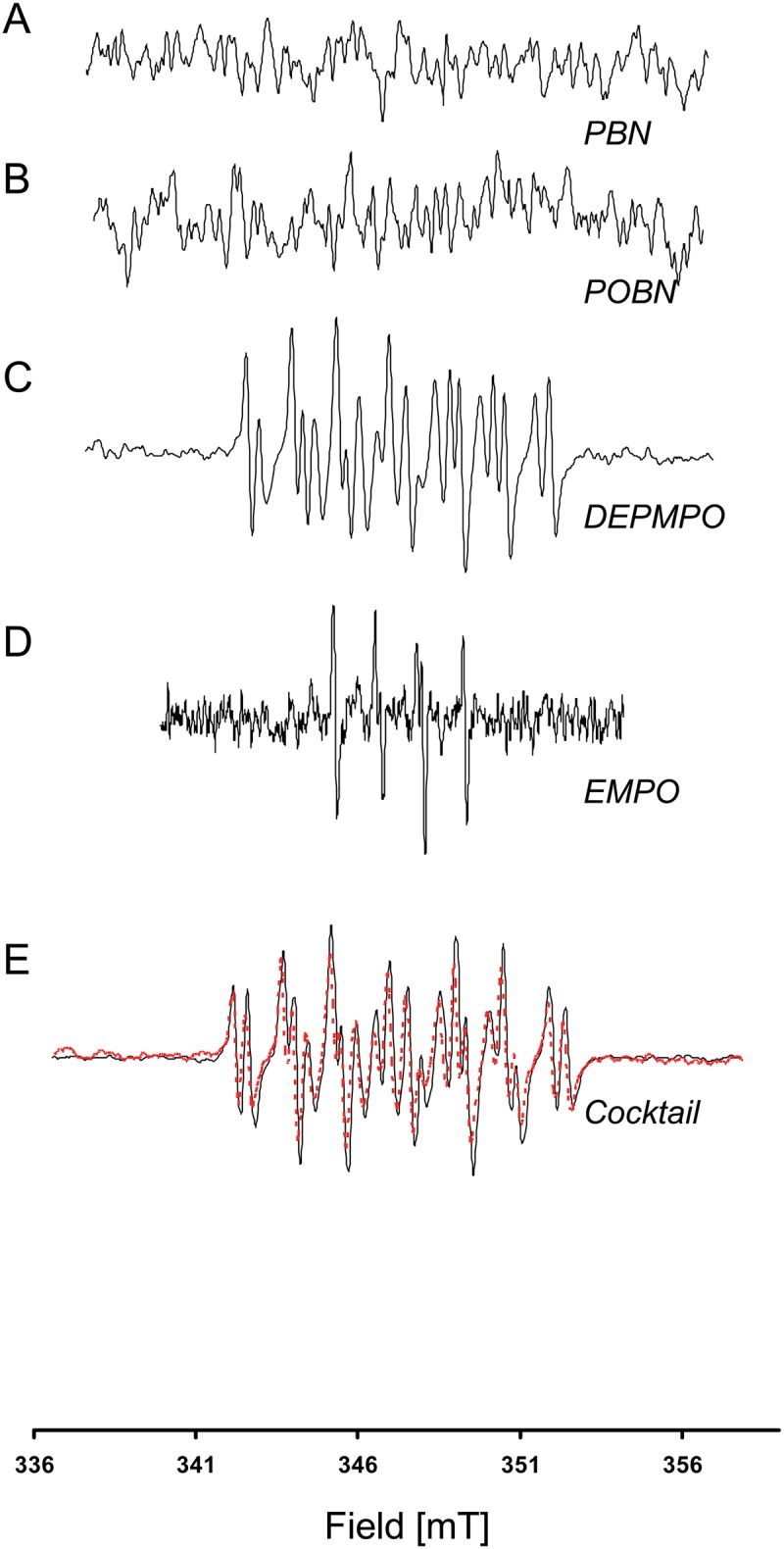
EPR spectra of K562 cells after exposure to menadione using individual spin trapping agents. A: PBN, B: POBN, C: DEPMPO, D: EMPO, E: Cocktail of spin traps (black) which is actually the sum of EPR spectra recorded using DEPMPO and EMPO (red dots).

To test whether our method could be applied to screening of compounds for their possible radical-mediated toxicity (the most likely application for this type of assay), we exposed non-adherent K562 cells to several known free-radical mediated toxics agents: tert-butylhydroperoxide, menadione, H_2_O_2_, and phenylhydrazine. For each compound, an EPR signature was recorded, confirming the involvement of a free radical reaction (Fig 4). To illustrate the potential interest of this approach, the particular case of menadione is presented in Fig 5. An EPR fingerprint was recorded using the cocktail of spin traps, whereas when some spin traps were used alone (here, PBN and POBN), they were unable to detect free radical involvement after menadione exposure (Fig 5).

This observation illustrates that false negatives can be produced when using traditional spin trapping with inadequate individual spin traps, and shows the potential appeal of using multiple spin traps with different properties in order to increase the chance of detecting free radicals in complex biological media.

Nevertheless, our method might suffer from a limitation due to the different kinetics of reaction between the different free radicals present in the medium and the different spin traps used, on the one hand, and the different rate of reduction of the radical-adduct formed into an EPR silent species, on the second hand. Indeed it can not be ruled out that a free radical would react preferentially with a specific ST, because of a very large constant of reaction, and simultaneously that the adduct formed would subsequently be very rapidly reduced into an EPR silent species. If the kinetic of reaction is sufficiently different, the free radical would not react at all with the other spin traps, and because of the reduction of the radical-adduct, no EPR signal would be recorded, leading to a false negative.

No signal was observed when the concentration of menadione was 0.5 mM; at a concentration of 2 mM, EPR spectra showed similar hyperfine splitting features and comparable intensities (data not shown). Higher concentrations were not tested in order to keep the mortality of cells below 10%. In the limited range of doses tested, a threshold effect was noticed but no formal dose effect. This could be explained because the spin trapping experiment measures the ROS signal at a definite time point that does not reflect the evolution of ROS production over the entire window of exposition to the toxic agent. The methodology is not suitable to detect an overall increase of ROS over time, and the increase of ROS at a particular time point is probably not large enough to be detected quantitatively by EPR spin trapping.

**Fig 6:**
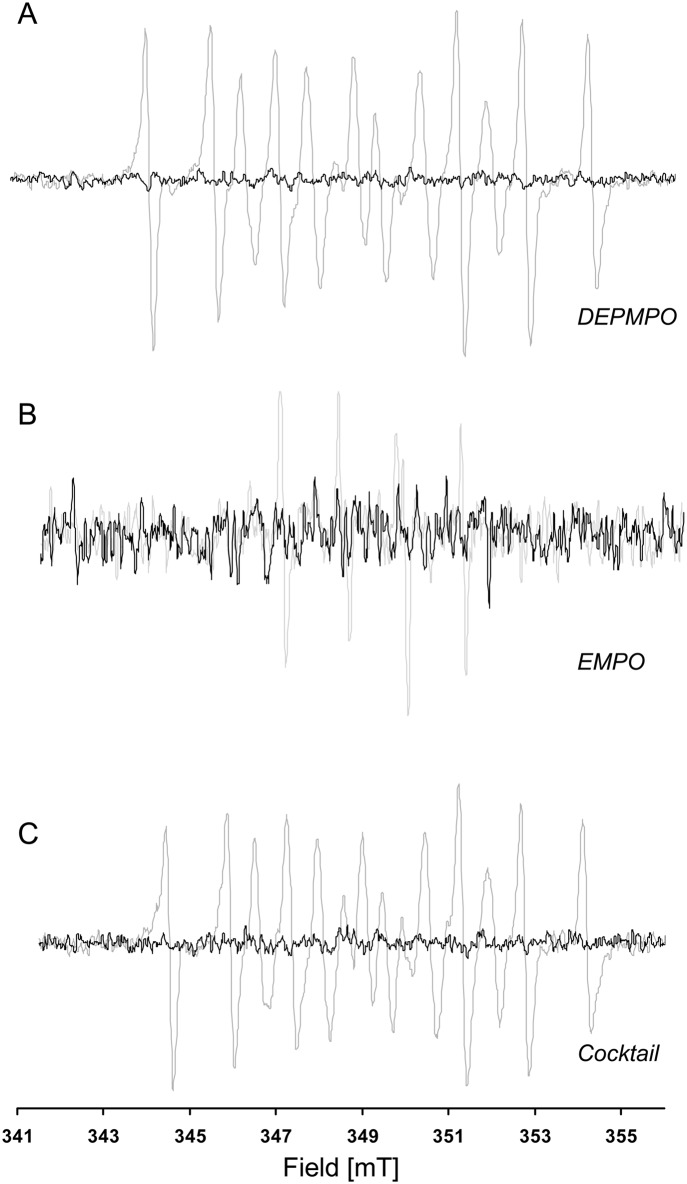
Influence of superoxide dismutase on the EPR spectra recorded after exposure to menadione. EPR spectra of a preparation of K562 cells (2x10^6^cells/ml) exposed to menadione (1 mM): A. in presence of DEPMPO (50 mM) (gray) or of DEPMPO (50 mM) + PEG-SOD (100U/ml) (black); B. in presence of EMPO (50 mM) (gray) or of EMPO (50 mM) + PEG-SOD (100U/ml) (black); C. in presence of cocktail (50 mM/spin trap) (gray) or of cocktail(50 mM) + PEG-SOD (100U/ml) (black).

Interestingly, the signal recorded after menadione exposure was a combination of the EPR signals recorded using DEPMPO and EMPO, with no particular distortion or alteration of the resultant spectrum (Fig 5). This finding demonstrates that the apparently complex EPR signal obtained using the cocktail of spin traps can be deconvoluted using an appropriate software to recover the individual components and, therefore, to point to the likely free radical involved, hence reducing the number of experiments that needs to be carried out to characterize free radicals produced in biological media. Finally, as it is well-recognized by researchers involved in spin trapping experiments, the final proof for the involvement of a particular free radical relies on the ability of specific enzymes to degrade the radical formed and abolish the EPR signature. This aspect is illustrated by the decrease in the EPR signal for cells exposed to menadione in the presence of PEG-SOD (Fig 6).

## Conclusion

In summary, the use of a cocktail of spin traps (differing in their reactivity and lipophilicity) is proposed as a screening tool that enable detection of a free radical process in biological media. The unique signature of the recorded EPR signal is a fingerprint of the free radical produced. As for other “-omics” techniques, this tool should be considered as a first screening test without *a priori* information, which will rapidly direct the biologist or the toxicologist to the likely radical species involved so that confirmatory tests can be conducted. Just as microarrays indicate which RT-PCR test needs to be performed to confirm the involvement of a particular gene, radicalomics will help in suggesting which assay needs to be performed to identify the free radical. Our proof-of-concept study should foster future research to test and compare the present assay with other combination of spin traps, to define optimal time-window for EPR detection in order to render this type of approach compatible with high-throughput screening in different biomedical disciplines (e.g., toxicology, biomaterial and drug development, or fundamental studies on oxidative stress).

## Supporting information

S1 Fig Spin trapping using a azide/peroxidase/H2O2 peroxidising system (azidyl radical formation). EPR spectra of phosphate buffer solution (pH = 7.4, 100 mM) with DTPA (10 μM), H_2_O_2_ (350 μM), horesedarish peroxidase (1mg/ml) and spin trap (20 mM) POBN (A), PBN (B), DEPMPO (C), EMPO (D) or the cocktail (E).
(TIF)

S2 Fig Spin trapping using a sodium sulfite/ sodium dichromate system (sulfite radical formation). EPR spectra of phosphate buffer solution (pH = 7.4, 100 mM) containing sodium sulfite (20 mM), sodium dichromate (10 mM) and 25 mM of spin trap POBN (A), PBN (B), DEPMPO (C), EMPO (D) or the cocktail (E).
(TIF)

S3 Fig Spin trapping using a Fenton system +10% (v/v) DMSO (methyl radical formation). EPR spectra of phosphate buffer solution (pH = 7.4, 100 mM) with DTPA (1 mM), Fe (NH^4^)_2_(SO_4_)_2_ (2m M), H_2_O_2_ (2 mM), 10% (v/v) DMSO and spin trap (25 mM) POBN (A), PBN (B), DEPMPO (C), EMPO (D) or the cocktail (E).
(TIF)

S4 Fig Spin trapping using a Fenton system (hydroxyl radical formation). EPR spectra of phosphate buffer solution (pH = 7.4, 100 mM) with DTPA (1 mM), Fe (NH^4^)_2_(SO_4_)_2_ (2m M), H_2_O_2_ (2 mM), and spin trap (25 mM) POBN (A), PBN (B), DEPMPO (C), EMPO (D) or the cocktail (E).
(TIF)

S5 Fig Spin trapping using a xanthine/xanthine oxidase system (anion superoxide radical formation). EPR spectra of phosphate buffer solution (pH = 7.4, 100 mM) containing xanthine (1mM), xanthine oxidase, DTPA (500 μM) and spin trap (25 mM) POBN (A), PBN (B), DEPMPO (C), EMPO (D) or the cocktail (E).
(TIF)
