# Supplementary figures and images for: Use of a cocktail of spin traps for fingerprinting large range of free radicals in biological systems
Source: PLoS One. 2017 Mar 2;12(3):e0172998. doi: 10.1371/journal.pone.0172998 (PMC5333873; doi:10.1371/journal.pone.0172998)

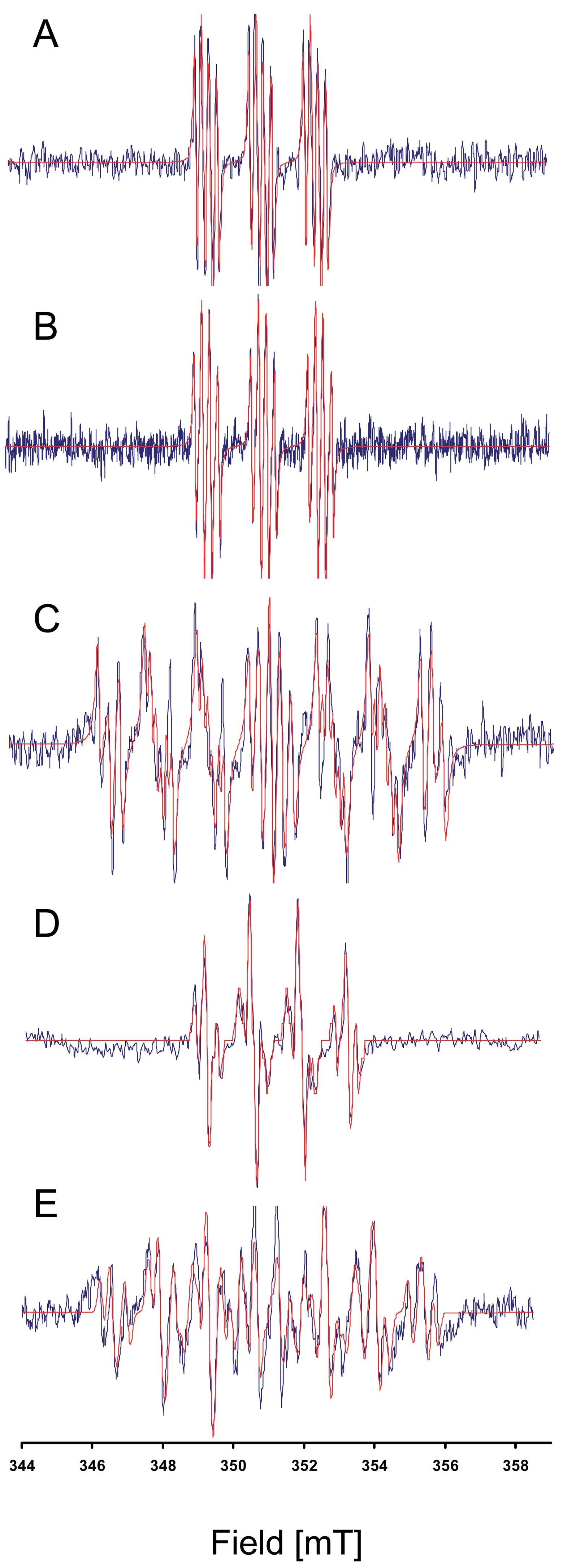

Supplement: S1 Fig — EPR spectra of phosphate buffer solution (pH = 7.4, 100 mM) with DTPA (10 μM), H2O2 (350 μM), horesedarish peroxidase (1mg/ml) and spin trap (20 mM) POBN (A), PBN (B), DEPMPO (C), EMPO (D) or the cocktail (E). (TIF) [file pone.0172998.s001.tif]

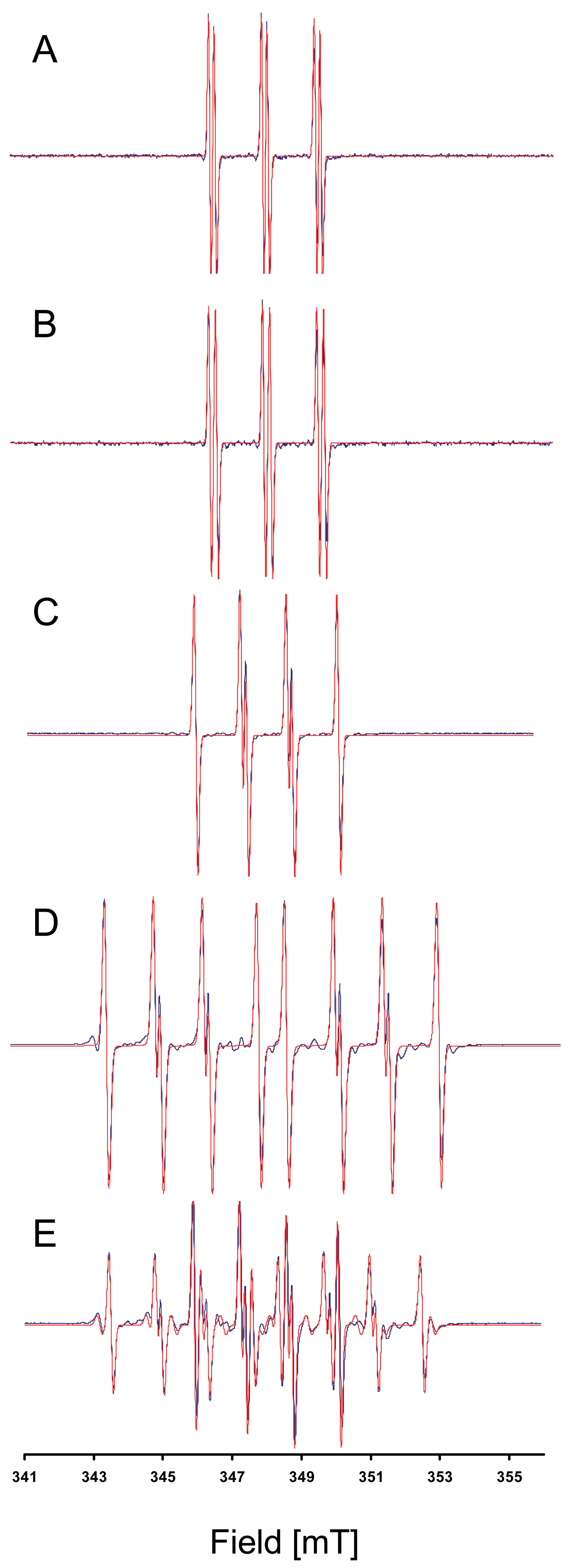

Supplement: S2 Fig — EPR spectra of phosphate buffer solution (pH = 7.4, 100 mM) containing sodium sulfite (20 mM), sodium dichromate (10 mM) and 25 mM of spin trap POBN (A), PBN (B), DEPMPO (C), EMPO (D) or the cocktail (E). (TIF) [file pone.0172998.s002.tif]

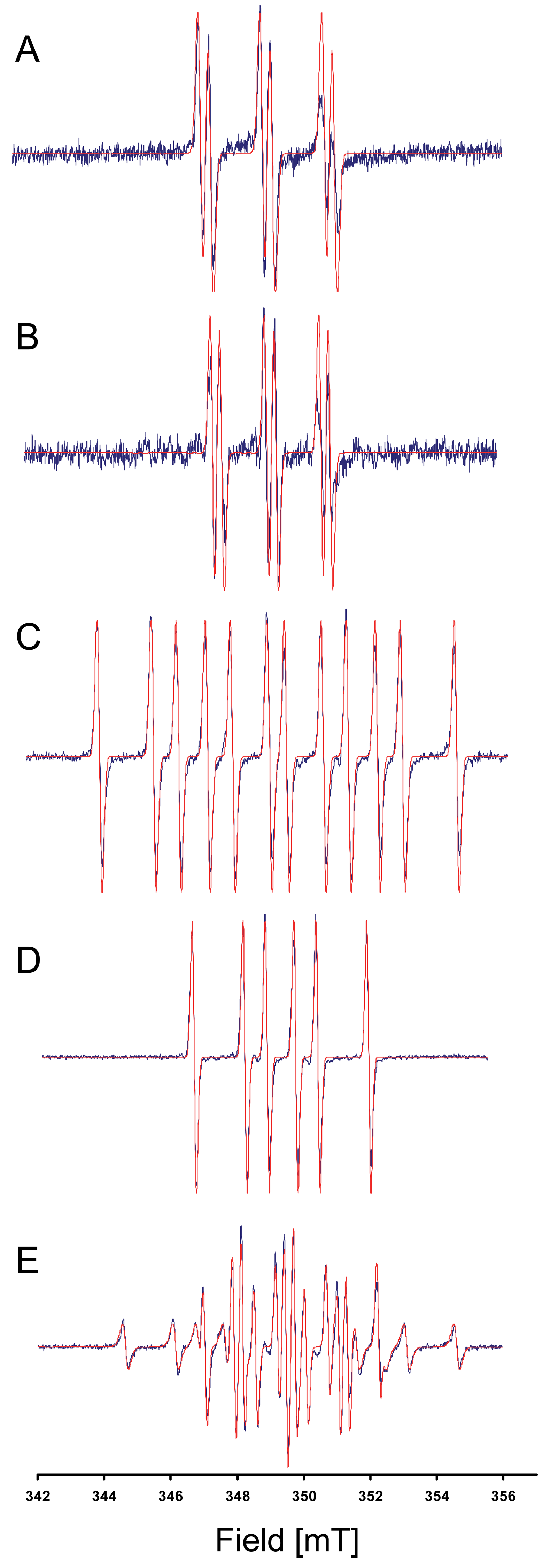

Supplement: S3 Fig — EPR spectra of phosphate buffer solution (pH = 7.4, 100 mM) with DTPA (1 mM), Fe (NH4)2(SO4)2 (2m M), H2O2 (2 mM), 10% (v/v) DMSO and spin trap (25 mM) POBN (A), PBN (B), DEPMPO (C), EMPO (D) or the cocktail (E). (TIF) [file pone.0172998.s003.tif]

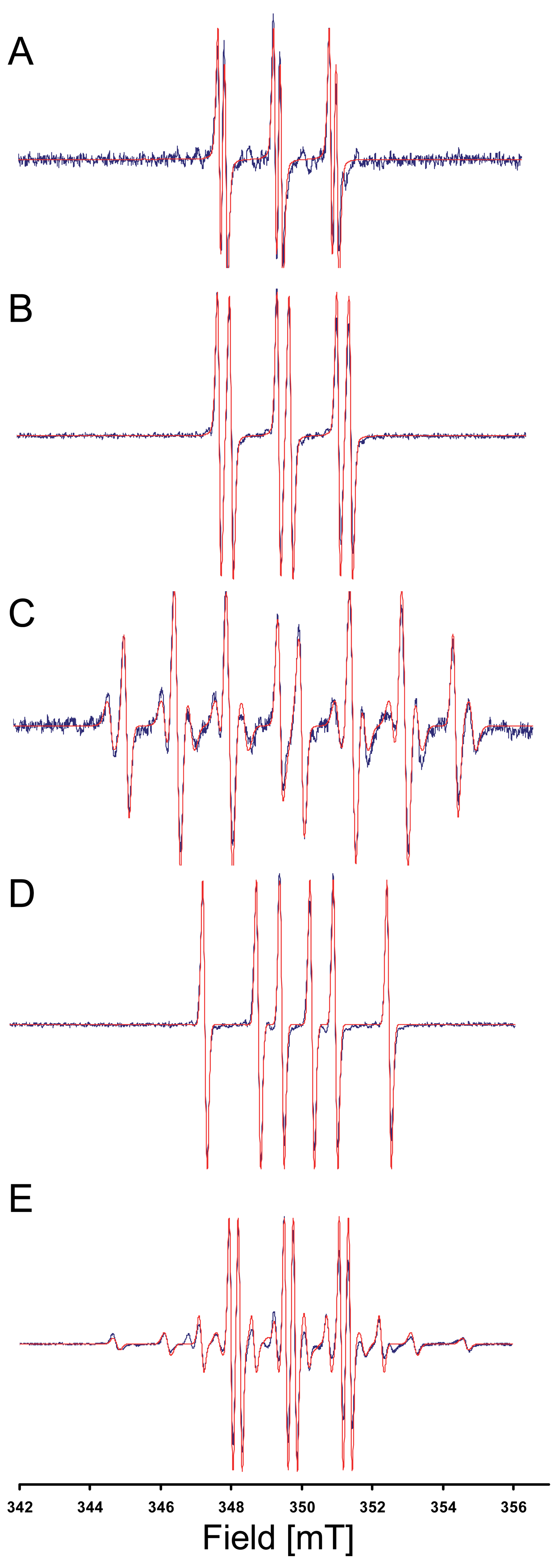

Supplement: S4 Fig — EPR spectra of phosphate buffer solution (pH = 7.4, 100 mM) with DTPA (1 mM), Fe (NH4)2(SO4)2 (2m M), H2O2 (2 mM), and spin trap (25 mM) POBN (A), PBN (B), DEPMPO (C), EMPO (D) or the cocktail (E). (TIF) [file pone.0172998.s004.tif]

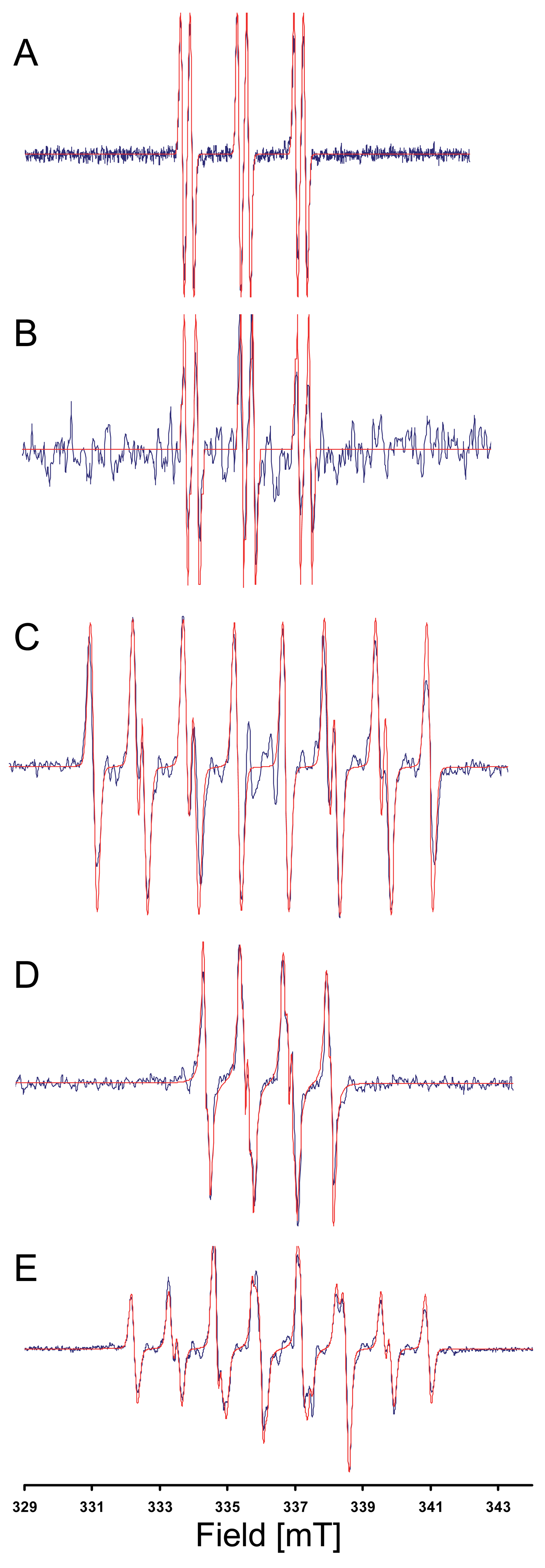

Supplement: S5 Fig — EPR spectra of phosphate buffer solution (pH = 7.4, 100 mM) containing xanthine (1mM), xanthine oxidase, DTPA (500 μM) and spin trap (25 mM) POBN (A), PBN (B), DEPMPO (C), EMPO (D) or the cocktail (E). (TIF) [file pone.0172998.s005.tif]
